# Extraperitoneal bladder rupture with severe lacerations of the urogenital diaphragm: a case report

**DOI:** 10.1186/1757-1626-2-56

**Published:** 2009-01-15

**Authors:** Akiyoshi Hagiwara, Koichiro Nishi, Keiichi Ito, Musasi Tobe, Tomohiko Asano, Masamichi Hayakawa

**Affiliations:** 1 Department of Traumatology and Critical Care Medicine, National Defense Medical College, 3-2 Namiki, Tokorozawa, Saitama, 359-8513 Japan; 2 Department of Urology, National Defense Medical College, 3-2 Namiki, Tokorozawa, Saitama, 359-8513 Japan

## Abstract

**Background:**

A 65-year woman injured by automobile had an open-book type pelvic fracture and extraperitoneal bladder rupture.

**Case presentation:**

Non-surgical management was selected because of suspected minor leakage. A follow-up CT cystography on day 21 showed a large urinoma. In the sagittal reconstruction CT images, the bladder was displaced downwards. Surgery was performed. Surgical findings showed that the bladder neck and the urethra were not anchored because of a laceration in the urogenital diaphragm. The bladder base had descended to the inferior margin of the pubic bone and a laceration of about 3 cm was found in the anterior surface of the bladder neck. The laceration was sutured, and the wall of the urinoma was extensively resected. The postoperative course was uneventful.

**Conclusion:**

An extraperitoneal bladder rupture associated with a severe laceration in the urogenital diaphragm will be indicated for surgery when the bladder is not anchored and healing is prevented. Sagittal reconstruction CT cystography was effective for this diagnosis.

## Background

Traumatic bladder rupture is a complication in 5 to 10% of pelvic fractures and 50 to 85% of such ruptures are extraperitoneal [1,2]. Treatment of extraperitoneal bladder rupture is generally conservative with only drainage and infection control. Spontaneous healing usually occurs in about 80% of patients in 10 to 14 days. Patients indicated for surgery are very rare because almost all of them show spontaneous improvement [3,4]. We experienced a surgical case of extraperitoneal bladder rupture associated with an open-book type pelvic fracture and a refractory urinoma extending from the anterior surface of the pubic bone to the medial side of the left thigh. This case is reported and surgical indications for extraperitoneal bladder ruptures are discussed.

## Case report

A 65 year-old woman was injured when an automobile pushed her against a concrete wall. Vital signs were normal but a pelvic fracture was suspected because of severe pain in the lumbar region and she was brought to the critical care center.

Her consciousness was clear and hemodynamics stable. Severe pressure pain was present on the anterior surface of the pubic bone and near the right sacrum. Macroscopic hematuria was found when a urethral catheter was inserted. A contrast enhanced CT (CECT) was performed on admission but the CT did not capture contrast leakage from the bladder. However, a pelvic X-ray post CT showed contrast leakage in the extraperitoneal space at the anterior of the bladder. An extraperitoneal bladder rupture was diagnosed. The open-book type pelvic fracture was treated with Hoffman's external fixation. Macroscopic hematuria was gradually improved and disappeared on day2.

**Figure 1:**
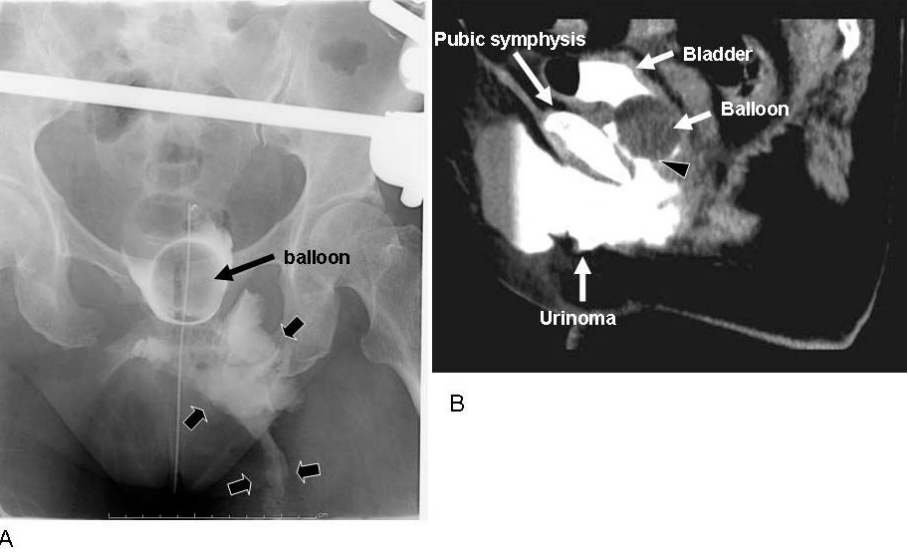
**Retrograde cystography (A) performed on day 4 and sagittal reconstruction image of CT cystography (B) performed later**. A: Leakage of extraperitoneal contrast medium outside the bladder is observed (arrows) B: In the sagittal reconstruction CT image, leakage of extraperitoneal contrast medium from the site shown by arrowheads is observed. The base of the bladder is positioned at the pubic bone inferior margin.

**Figure 2:**
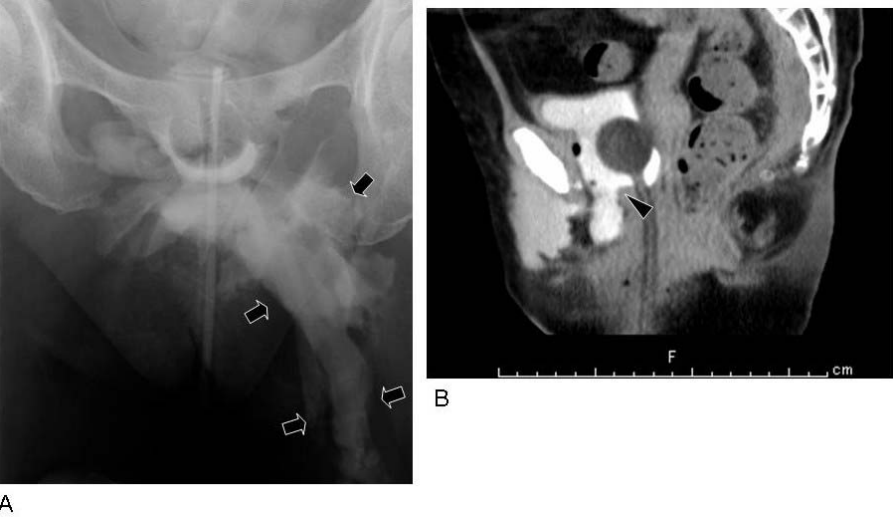
**Retrograde cystography (A) performed on day 16 and sagittal reconstruction image of CT cystography (B) performed later**. A: The urinoma extending to the left thigh is enlarged when compared with day 4 (arrows). B: In the sagittal reconstruction CT image, the laceration is more clearly defined (arrowhead). The base of the bladder is positioned at the pubic bone inferior margin lower than the image obtained on day 4.

On day 4, retrograde cystography (Fig. 1) and CT cystography were performed. An urinoma was found to extend from the anterior surface of the pubic bone to the medial side of the left thigh. In a sagittal reconstruction CT image, a leakage site of the contrast medium was observed at the lower anterior surface of the bladder. The base of the bladder had descended to the inferior margin of the pubic symphysis. The patient's general condition was very good and no symptoms of infections were found. Urine drainage through a urethral catheter was good. Therefore, we though that this urinoma was spontaneously discharged through a urethral catheter, and the bladder laceration to extraperitoneum was spontaneously healed. Non-surgical management was selected. CT cystography were performed on day 21. As shown in Fig. 2, the urinoma had become enlarged. The base of the bladder had descended to lower than the CT on day 4. Surgery was performed.

**Figure 3:**
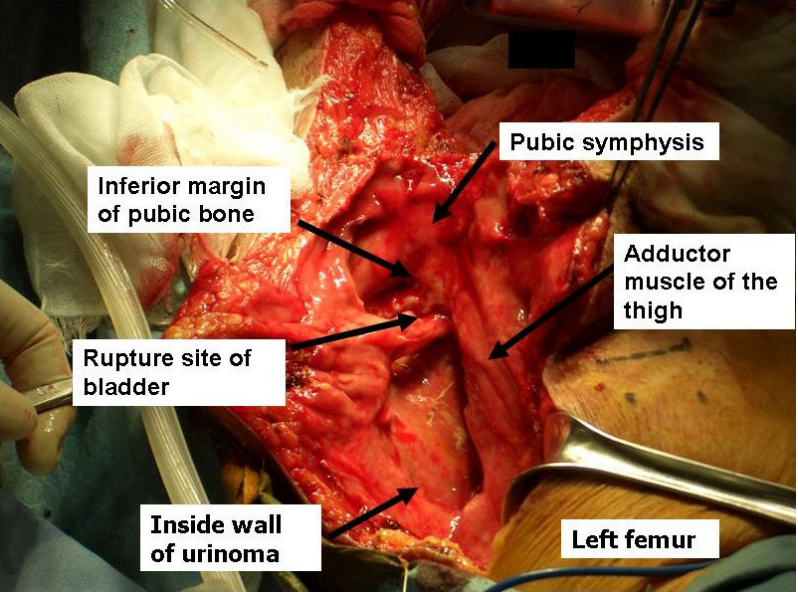
**Surgical findings**. A midline incision of the lower abdomen was made and the incision was extended through a front of the pubic symphysis to the left thigh. A bladder laceration was found in the inferior margin of the pubic symphysis. Partial laceration of the left femoral adductor muscle at the pubic insertion and an urinoma extending from the anterior surface of the pubic bone to the left thigh were observed. The wall of the urinoma had changed to glossy membranous tissue with a flat smooth surface.

A midline incision of the lower abdomen was made and the incision was extended through a front of the pubic symphysis to the left thigh. Surgical findings (Fig. 3) revealed that the bladder was not anchored because of severe lacerations in the urogenital diaphragm, and had a laceration of about 3 cm in the anterior surface of the bladder neck. The findings also showed a partial laceration of the left femoral adductor muscle at the pubic insertion and an urinoma cavity extending from the anterior surface of the pubic bone to the left thigh. The wall of the urinoma consisted of membranous tissue with a flat smooth surface. The laceration of the bladder was closed by sutures. The membranous tissue of the urinoma wall was partially resected and the remaining membranous tissue was extensively coagulated. Four suction drains were placed and the urinoma cavity was closed. The retropubic suspension procedure was also performed to prevent postoperative urinary incontinence. Sutures were placed on either side of the bladder neck, taking bites through the paravesical fascia and anterior vaginal wall. The most distal suture is placed at the level of the bladder neck. Each suture was then passed into an appropriate site in the cartilaginous portion of the symphysis.

Pathological findings of the urinoma wall showed histology with severe hyperplasia of collagen fiber and fibroblasts.

The postoperative course was uneventful. When cystography was performed on postoperative day 16, no leakage of the contrast medium was found. The urethral drain was removed 3 weeks postoperatively and the Hoffman's external fixation was removed on day 55. Urgent incontinence was seen one time just after the catheter removal and urinary incontinence was not seen thereafter. She was discharged on foot on day 65. Neurogenic bladder did not occur during follow-up period.

## Discussion

Surgical indications for extraperitoneal bladder ruptures include concomitant injury to the vagina or rectum, injury to the bladder neck in females or children, avulsion of the bladder neck, or cases with a foreign body in the lumen of the bladder such as a bone spicule from a pelvic fracture [5]. However, almost all patients with extraperitoneal bladder rupture show spontaneous healing in about 2 weeks when they do not have these injuries [3].

In our case, spontaneous closure had not occurred after 2 weeks. The reasons for this appeared to be that (1) the anterior surface of the bladder was not anchored because of the laceration in the urogenital diaphragm and closure did not occur at the site of the laceration, and (2) a laceration of the femoral adductor muscle allowed urinoma formation in the medial side of the thigh.

Spontaneous closure might be achieved by direct drainage of the urinoma, but the inside wall of the urinoma had changed to membranous tissue with hyperplasia of collagen fiber and fibroblasts. After these changes occurred, spontaneous closure with drainage alone will be difficult.

Patients with pelvic fractures caused by severe frontal external force are susceptible to lacerations of the urogenital diaphragm, especially the pubovesical muscle. We believe that such lacerations cause loss of anchoring of the bladder, and spontaneous healing of extraperitoneal bladder ruptures will be suppressed. It is difficult to diagnose an injury of the urogenital diaphragm by routine CT images but in sagittal reconstruction CT images, the base of the bladder had descended to the inferior margin of the pubic symphysis (Figs 1 and 2). These findings will show a loss of anchoring of the bladder.

We believe that surgical indications of extraperitoneal bladder ruptures include not only the indication criteria given in the literature, but also loss of anchoring of the bladder due to severe laceration of the urogenital diaphragm. Such lacerations can be diagnosed by sagittal reconstruction of CT images.

## Consent

Written informed consent was obtained from the patient for publication of this case report and accompanying images. A copy of the written consent is available for review by the Editor-in-Chief of this journal.

## Competing interests

The authors declare that they have no competing interests.

## Authors' contributions

AH and KN were attending physicians of this patient and prepared the manuscript. KI, MT, and TA performed the surgery and conceived the study. MH edited and coordinated the manuscript. All authors read and approved the final manuscript.
